# Rapid Prototyping of Pneumatic Directional Control Valves

**DOI:** 10.3390/polym13091458

**Published:** 2021-04-30

**Authors:** Slawomir Blasiak, Pawel Andrzej Laski, Jakub Emanuel Takosoglu

**Affiliations:** Faculty of Mechatronics and Mechanical Engineering, Kielce University of Technology, Aleja Tysiaclecia Panstwa Polskiego 7, 25-314 Kielce, Poland; sblasiak@tu.kielce.pl (S.B.); qba@tu.kielce.pl (J.E.T.)

**Keywords:** additive manufacturing, polymer materials, pneumatic valve, rapid prototyping, PolyJet Matrix

## Abstract

The main objective of the study was to design a pneumatic directional control valve for controlling pneumatic drives and produce it using a rapid prototyping technique. As the basic design assumption was to achieve high performance through a high flow rate and a low pressure drop, it was necessary to determine two flow parameters: the sonic conductance and the critical pressure ratio. The flow rate of compressed air and the diameters of the pneumatic conduits and fittings are important as they affect the rate of travel of the pneumatic cylinder piston. The 3D solid model of the directional control valve, developed in a CAD program, was used to simulate and optimize the flow rate. The analysis was performed by means of ANSYS CFX, a computational flow dynamics program. The main elements of the valve, i.e., the spool and the body, were produced using the PolyJet Matrix technology. The prototype was tested experimentally to determine the nominal flow-rate, calculate the flow parameters in accordance with the ISO 6358-1989 standard and compare them with the CFD simulation data. The simulation results showed very good agreement with the measurement data. The CFD analysis of the 3D solid model enabled us to optimize the flow of compressed air through the valve. The rapid prototyping method was found to be suitable to produce a fully functional directional control valve, which was confirmed through measurements at a test stand. The attempt to combine rapid prototyping used to fabricate pneumatic directional control valves with CFD used to simulate their operation was successful. The study shows that it is possible to design and construct a fully functional directional control valve characterized by high efficiency, high performance and a small pressure loss in a very short time and at a very low cost, which makes rapid prototyping superior to conventional methods of prototype making.

## 1. Introduction

Rapid prototyping (RP) can be defined as creating, layer by layer, physical three-dimensional objects from 3D computer-aided design data. Although the term rapid prototyping is commonly used, it is not precise. The technology is not limited only to the production of prototypes; it is adequate to fabricate both fully functional finished products and tools. The applications of rapid prototyping range from industrial to architectural to archaeological. Rapid prototyping of physical models is also becoming increasingly more important in medicine (e.g., implantology, prosthetics, forensic medicine).

Kheirollahi [1] analyzes the use of RP in dentistry, especially the RP methods for the design and fabrication of dentist prostheses. In another major study concerned with the application of RP, Soe et al. [2] discuss the possibility of reconstructing elements of a medieval ship. They also attempt to assess the degree of applicability of additive manufacturing to reconstruct other archaeological finds and indicate problems related to the application of reverse engineering and rapid prototyping. Lantada [3] focuses on a relevant issue, namely further development of rapid prototyping for the purposes of biomechanics, biomedical engineering and medical sciences [4,5,6,7,8].

Today, mechanical components are commonly developed using finite element methods combined with topology optimization to obtain the required dimensions. Elements optimized in this way generally have irregular shapes, difficult to produce with methods other than RP. Gardan and Schneider [9] describe the product creation process involving the use of RP and topology optimization.

Santana et al. [10] analyze the application of RP methods to produce 3D printed mesh reinforcement for brittle materials. In their study, they reported a considerable increase in the fracture toughness of geopolymer composites with such reinforcement when these were subjected to bending loads.

In [11], Hopkinson and Dickens provide an in-depth analysis of RP, aiming at the identification of the potential of this technology and its costs. Their research focuses on the properties of materials, quality control and usability of products.

Despite being a relatively new technique, rapid prototyping is commonly used for industrial and everyday purposes as well as in highly specialized areas of science. This is mainly due to the fact that the technology is extremely flexible; it can be used for producing elements that would be difficult or even impossible to make using conventional methods. It is also important that the fabrication time is relatively short and the functionality of a product can be verified immediately [12]. If errors are detected or the quality is unsatisfactory, the product is redesigned with no additional costs; this is not possible for products made by casting or injection molding.

There are many 3D printing technologies currently available in the market, each using specific materials. Selective Laser Sintering (SLS) is one of the most popular. Since it does not require support structures, it is well-suited for manufacturing elements that need to be mechanically strong, are complex in shape and design or will operate in sliding contact. SLS is generally used in short series production or to fabricate customized products. It is also commonly employed to test and validate form, fit and functionality, which is essential for automotive, aerospace, medical, machine or appliance parts [13,14]. Stereolithography (SLA, or SL) is the oldest 3D printing technology and still the most common for rapid prototyping. It is a laser-based method in which liquid resin is exposed to UV radiation. An object is printed “bottom up” by focusing a UV laser beam to scan the resin surface and selectively cure the material as successive cross-sectional layers are built. Supports required for protruding elements or cavities are formed automatically; their removal, however, is manual. SLA has a wide range of applications including visual prototypes, display elements, vacuum-casting (VC) patterns, industrial and non-industrial design projects, investment casting molds (TetraShell) and elements with intricate details. As it enables surface metallization, it is also an alternative for sheet metal prototypes [15,16].

Another popular 3D printing method is Fused Deposition Modeling (FDM), which involves depositing successive layers of thermal plastic. Deposition of melted material resembles printing with an ordinary ink jet printer. Heated nozzles moving along the X and Y axes melt the material to form a 3D object on the platform, which is lowered after each layer in the Z direction. This technique offers ready-to-use elements for the automotive and aviation industries; it is also suitable for prototyping in a variety of industries. The key advantage of FDM is the durability of materials, high quality of objects and stability of their mechanical properties over time. The materials employed in this technology are, for instance, e.g., ABS, [17,18]. Selective Laser Melting (SLM), or 3D metal printing, is becoming increasingly popular. Direct printing to obtain complex, end-use products with high surface finish optimizes costs, which would be higher in traditional methods of production; it also reduces the object mass and the lead time. Like in Selective Laser Sintering (SLS), parts are built from metal powder deposited in thin successive layers and then exposed to laser light. Support structures of the same material are created automatically, but they need to be removed manually. Printed objects undergo heat treatment. There is a high degree of flexibility of design in terms of mechanical properties of metal [19].

HP’s Multi Jet Fusion (MJF) technology is well-suited for processes where short lead times, low porosity and high surface quality are required. Multi Jet Fusion is a powder-based technology with no use of laser. First, the powder bed is uniformly heated. Then, the fusing agent is sprayed onto the material to fuse particles selectively. The detailing agent is sprayed around the contours to improve the object resolution. When the lamps pass over the surface of the powder bed, the sprayed material absorbs heat and helps distribute it evenly. Multi Jet Fusion uses fine-grained materials to produce ultra-thin layers, 80 micron in thickness. Objects obtained in this way have higher density and lower porosity than those printed by selective laser sintering. The smooth surface achieved by the printer requires minimum finishing. This implies shorter lead times, which are crucial with functional prototypes and in small series manufacturing. The materials common in this technology are PA 12 and Ultrasint TPU 90A-01 [20,21].

Objects printed by PolyJet printing may differ in color, material, as well as mechanical and physical properties. PolyJet is the first technology that allows two types of resin to be deposited simultaneously in one process. It can thus be applied to produce complex high-quality objects. PolyJet involves spraying photocurable polymer to form an ultra-thin layer on the build tray. Each photopolymer layer is cured with UV light just after it is sprayed, which means printed objects can undergo finishing immediately. After 3D printing, gel-like support material is removed by spraying it with water. The PolyJet technology can be used to form horizontal layers with a thickness of just 16 microns, tiny details, and ultrathin 0.6 mm thick walls, depending on the object geometry. PolyJet printing is commonly used to fabricate high precision mechanical subassemblies, components with elastic properties (seals, gaskets, O-rings, washers), components in a range of Shore A hardness scale values, casting master patterns (e.g., in vacuum casting) and high-quality prototypes within short lead times. The materials used in the PolyJet technology are objet Vero colored, objet VeroClear, objet Tango, and composites [17,22,23].

From a review of the literature it can be concluded that the application of rapid prototyping is limited only by our imagination. The potential of the RP technology is so high that it seems suitable also for producing prototypes of parts of, or whole pneumatic directional control valves. In this study, the directional control valves fabricated by additive manufacturing were assessed through simulations and experiments and the results confirm that the process is well-suited for this purpose.

The method proposed here is well-suited to develop prototypes of directional control valves for various industrial applications. It helps significantly reduce costs and time required to carefully design and then manufacture different types of valves. Verification of CFD simulation results obtained for 3D printed valve models, which requires comparing them with experimental data, is of crucial importance, as it allows us to discover and eliminate design errors at early stages, and, if necessary, easily redesign the valve before it reaches the production stage. This approach to the development of pneumatic directional control valves can be viewed as innovative.

## 2. Valve Model

**Figure 1:**
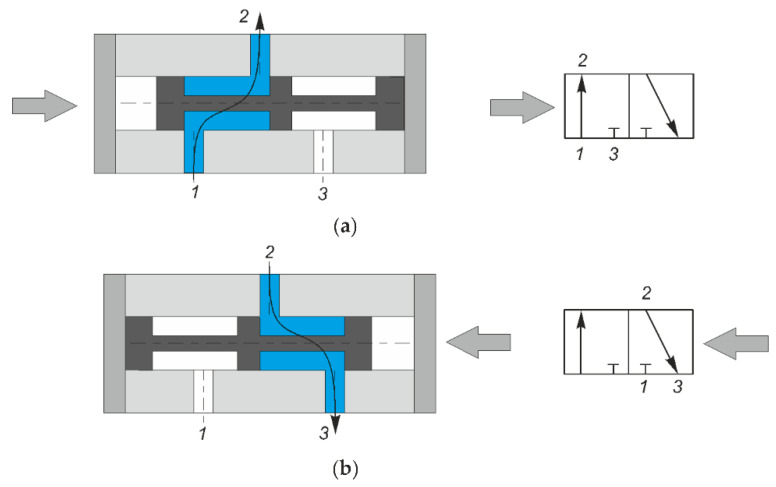
Diagram of the 3/2 valve (**a**) fluid flowing along ways 1 and 2, with way 3 being cut off (**b**) fluid flowing along ways 2 and 3, with way 1 being cut off.

Pneumatic directional control valves are elements designed to control the fluid flow in pneumatic drive and control systems [24,25]. The direction of the fluid flow in a directional control valve can be changed by moving the spool. The spool is generally a plunger with seals, moving inside the body of a directional control valve between two extreme positions. Figure 1 shows a diagram of the 3/2 valve with its principle of operation. 3/2 directional control valves are some of the most common sliding spool valves. When the spool is on the right (Figure 1a), the fluid flows along ways 1 and 2, with way 3 being cut off. After the spool moves to the left (Figure 1b), the fluid flows along ways 2 and 3, with way 1 being cut off.

**Figure 2:**
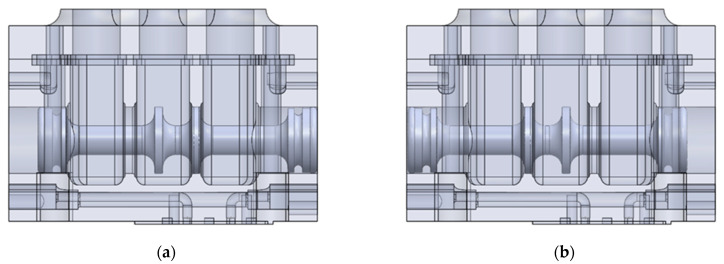
Solid model of the 3/2 valve, (**a**) fluid flowing along ways 1 and 2, with way 3 being cut off (**b**) fluid flowing along ways 2 and 3, with way 1 being cut off.

Figure 2 shows the solid model of the 3/2 directional control valve developed in a 3D CAD program, taking into account the above principle of operation and the information about the existing designs.

Pneumatic directional control valves fall into directly and indirectly actuated. In the valve considered here, control signals come from the control elements. In practice, these are miniature pneumatic valves with electromagnetic control and low power of an order of several watts. Such a solution is economically beneficial, because if the spool were actuated in a direct way by means of electromagnets, considerable power would be necessary to produce a force resisting the motion of the spool and the pressure of the compressed air acting on the spool. Indirect actuation of directional control valves involves switching an electric or external pressure signal of the adaptor (most commonly, a mini valve) to ensure adequate pressure of the working fluid on the active surface of the spool of the main valve, causing its movement. Generally, the initial valve is controlled mechanically as well. This allows the valve spool to shift with no need to provide electric or pneumatic signals. The key advantage of indirect actuation is that it is possible to control valves with a high flow rate using small-power electromagnets [26]. The main drawback is that the spool shifting and idle times are longer, when compared with those reported for directional control valves.

**Figure 3:**
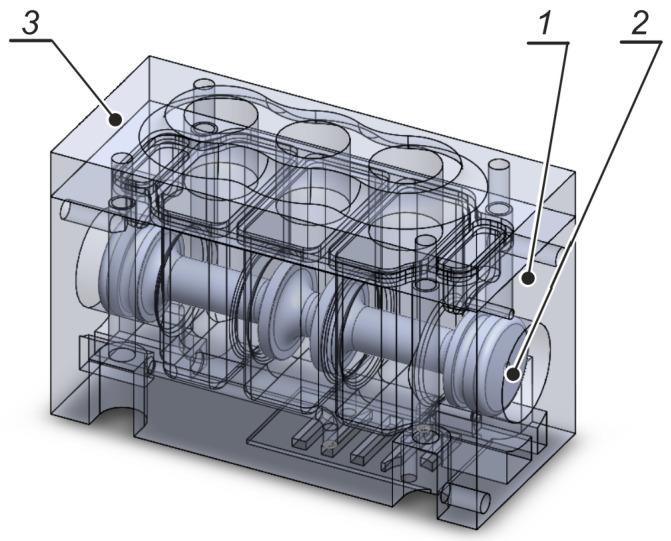
Solid model of the valve.

**Figure 4:**
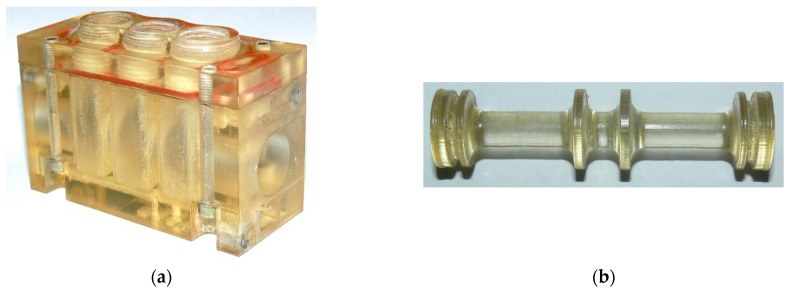
Physical model made using the PolyJet Matrix technology: (**a**) valve body and the subbase, (**b**) spool.

The drawing in Figure 3 illustrates the main elements of the valve: the body (1), the spool (2) and the subbase (3), whereas Figure 4 shows a general view of the prototype valve produced by additive manufacturing through RP.

This prototype will be assessed through simulations with the ANSYS Academic Research program and through experimental testing.

## 3. Mathematical Model

The analysis with methods of computational fluid dynamics (CFD) uses a transformation of differential equations of transport to find out more about the phenomena occurring in the valves.

The methods of computational fluid mechanics are an alternative to empirical studies, which are costly (sophisticated measuring devices) and extremely time-consuming. An advantage of the computational fluid mechanics methods is that the calculation results are independent of the process scale, provided that correct detail models are employed.

The computational process, based on the laws of fluid motion, involved creating a mathematical model of the directional control valve in the form of equations describing fluid flow physics.

The turbulent flow of a viscous fluid is described with Reynolds Equations (2)–(4), which, together with the continuum Equation (1) constitute a complete system of relationships to be used to calculate the pressure and the flow rate.

(1) $$\frac{\partial \rho }{\partial t}+div\left(\rho U\right)=0$$

Reynolds equations:

(2) $$\frac{\partial \left(\rho U\right)}{\partial t}+div\left(\rho UU\right)=-\frac{\partial P}{\partial x}+div\left(\mu \cdot gradU\right)+\left[-\frac{\partial \left(\rho \bar{{u^{\prime }}^{2}}\right)}{\partial x}-\frac{\partial \left(\rho \bar{u^{\prime }v^{\prime }}\right)}{\partial y}-\frac{\partial \left(\rho \bar{u^{\prime }w^{\prime }}\right)}{\partial z}\right]$$

(3) $$\frac{\partial \left(\rho V\right)}{\partial t}+div\left(\rho VU\right)=-\frac{\partial P}{\partial y}+div\left(\mu \cdot gradV\right)+\left[-\frac{\partial \left(\rho \bar{u^{\prime }v^{\prime }}\right)}{\partial x}-\frac{\partial \left(\rho \bar{{v^{\prime }}^{2}}\right)}{\partial y}-\frac{\partial \left(\rho \bar{v^{\prime }w^{\prime }}\right)}{\partial z}\right]$$

(4) $$\frac{\partial \left(\rho W\right)}{\partial t}+div\left(\rho WU\right)=-\frac{\partial P}{\partial z}+div\left(\mu \cdot gradW\right)+\left[-\frac{\partial \left(\rho \bar{u^{\prime }w^{\prime }}\right)}{\partial x}-\frac{\partial \left(\rho \bar{v^{\prime }w^{\prime }}\right)}{\partial y}-\frac{\partial \left(\rho \bar{{w^{\prime }}^{2}}\right)}{\partial z}\right]$$

Reynolds assumed that in the turbulent flow, all the characteristic parameters, including the flow rate and fluid pressure, could be represented as sums of the average values, that is the slow-varying parameters and turbulent fluctuations.

The *k-ε* model is one of many models with two additional equations proposed by Chou (1945) and then modified, for instance, by Davidov, Harlow and Nakayama, Jones and Laudner. Currently, it is one of the most popular models employed to simulate turbulent flow. As mentioned above, two parameters (*k-ε*) require additional equations of transport, which can be written as:

(5) $$\frac{\partial \left(\rho k\right)}{\partial t}+div\left(\rho kU\right)=div\left(\frac{\mu_{t}}{\sigma_{t}}\cdot gradk\right)+\mu_{t}\phi -\rho \varepsilon$$

(6) $$\frac{\partial \left(\rho \varepsilon \right)}{\partial t}+div\left(\rho \varepsilon U\right)=div\left(\frac{\mu_{t}}{\sigma_{t}}\cdot grad\varepsilon \right)+C_{1}\mu_{t}\frac{\varepsilon }{k}\phi -C_{2}\rho \frac{\varepsilon^{2}}{k}$$

where *k* denotes the turbulence kinetic energy, and *ε* is the turbulence kinetic energy dissipation.

The above differential partial equations were implemented in the computational module of the ANSYS CFX program. To effectively solve the system of equations describing the turbulent flow of the fluid, it was necessary to use boundary conditions that guarantee the uniqueness of the solution and affect the computational process for the area analyzed.

## 4. Simulations

**Figure 5:**
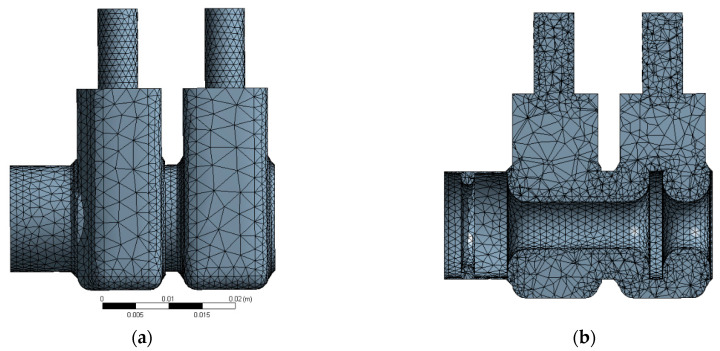
Valve model meshed with ANSYS: (**a**) general view (**b**) view of the cross-section through the axis of symmetry.

This section describes a numerical analysis of the flow of compressed air through the directional control valve. The solid model created with the ANSYS Academic Research program was used to form an internal space of the valve along ways 1 and 2, which is filled with the fluid. The next stage involved generating a spatial non-structural computational mesh (Figure 5) consisting of more than 46,500 triangular elements and 10,815 nodes. The mesh density was higher near the walls and around the spool, which was related to the character of the flow phenomena occurring near the non-moving elements of the valve. Another assumption was that the fluid flowing through the valve should had properties of an ideal gas.

The boundary conditions for the flow model were also determined. It was assumed that the inlet pressure along way 1 was 0.63 MPa, whereas the pressure along way 2 was equal to atmospheric pressure.

**Figure 6:**
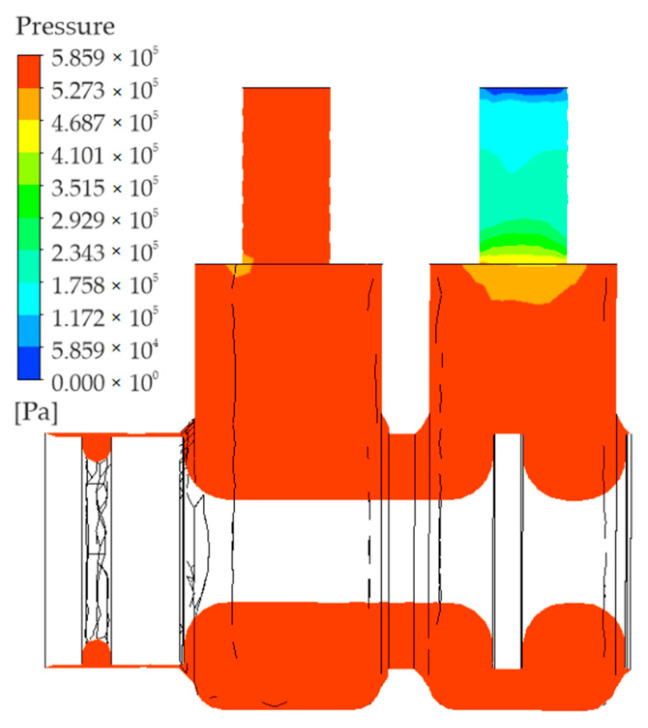
Distribution of pressure.

The ANSYS Academic Research (CFX) program was used to perform simulations with a view to determining the distribution of air pressure in the working volume. Figure 6 shows the graphical representation of the absolute pressure distribution in a plane perpendicular to the axis of symmetry of the valve.

As can be seen, the pressure is virtually stable and equal to the pressure at the inlet to the valve. Expansion takes place at the outlet; the pressure in the outlet port is almost equal to ambient pressure.

**Figure 7:**
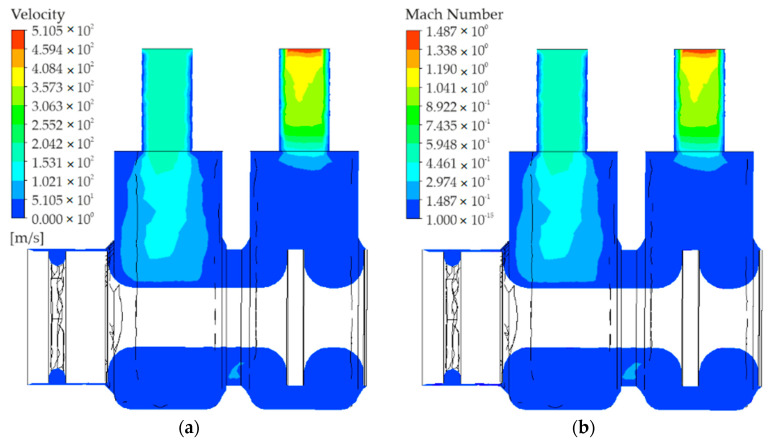
Distribution of the (**a**) gas flow rate and (**b**) Mach number.

Figure 7 shows the distribution of the gas flow rate (velocity) and the gas Mach number.

Air flows into port 1 with a rate of approximately 300 [m/s]. As can be seen, the rate with which air flows through the inlet and outlet close to the walls is equal to zero. The highest rate is achieved in the central part. Figure 7 shows that the rate of air flowing into way 2 increases from a value close to zero to over 510 [m/s], (i.e., almost 1.49 Mach) in the central part of the outlet (along way 2).

**Figure 8:**
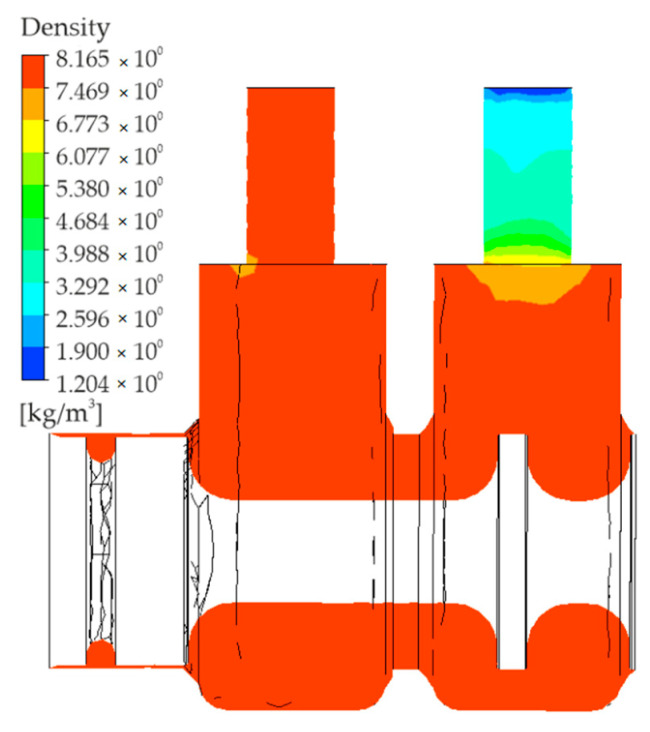
Distribution of density.

The density distribution diagram is very similar to the diagram provided in Figure 8. A minimum density of approximately 1.2 $\left[\mathrm{kg}/m^{3}\right]$ is observed at the end of way 2; it actually corresponds to the density of expanded air (atmospheric air). The air density in the remaining volume is generally stable and reaches a maximum of 8.16 $\left[\mathrm{kg}/m^{3}\right]$.

Simulations were performed to analyze the flow of compressed air through the directional control valve. The results were compared with the experimental data for the purpose of full verification.

## 5. Rapid Prototyping

**Figure 9:**
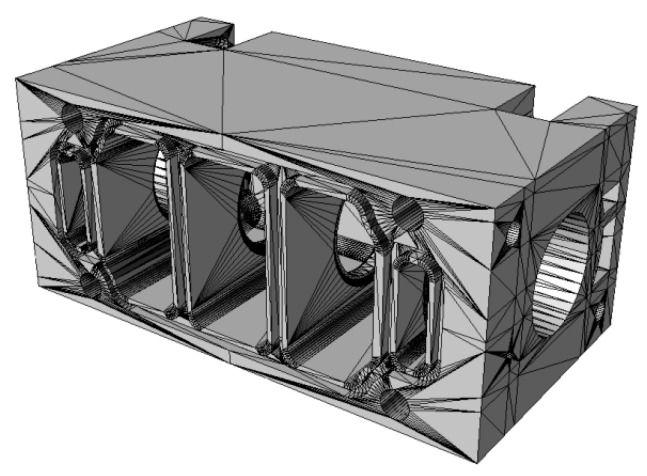
STL model.

The first step of the rapid prototyping process is to define an object as a 3D-CAD model, which is illustrated in Figure 3. The model is then numerically transformed into a set of data adequate for RP systems. During data handling, the CAD file is converted to the STL format, which uses triangles. Each of the triangles is described by a normal directional unit vector and three points constituting the triangle vertices. The STL model is shown in Figure 9.

STL, which is a universal file format, can be read by the program dedicated to the Connex 350 printer, i.e., Objet Studio.

**Figure 10:**
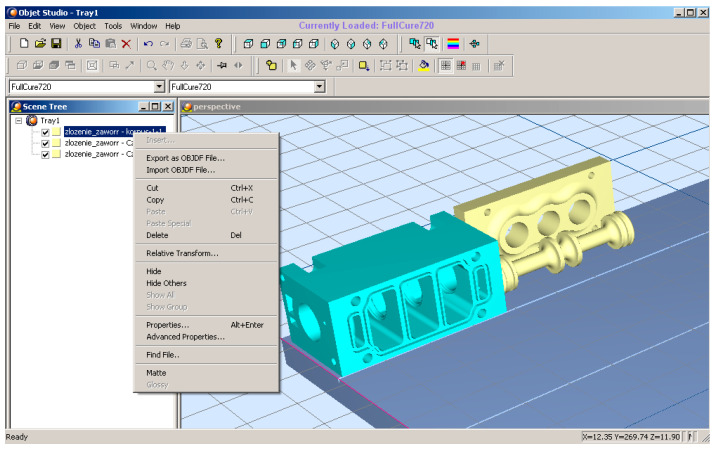
Objet Studio dialog box.

A general view of the Objet Studio dialog box is provided in Figure 10.

The input files are manipulated using Objet Studio. As a result, the elements are scaled, rotated and properly arranged on the built tray. The program enables us to select the material for each element to be created.

The PolyJet Matrix technology helps build physical models with different mechanical and physical properties in one printing process, which results from the simultaneous feeding and mixing of two types of resin, each having different properties. In this technology, the printing time is short, and the prototypes are precise.

In the case described here, the build material was resin with a commercial name FullCure720, which is a transparent material characterized by high stiffness.

After a model is oriented in the workspace, it is numerically sliced into layers. In the subsequent step, the data are sent to the printer and the fabrication process starts. A Connex 350 has three modes of printing: High Quality, High Speed and Automatic. The printing time depends on the mode, and so does the print accuracy. Highest accuracy models are obtained in the High Quality mode.

The program estimates the approximate time required to produce the elements and the approximate masses of the build and support materials to be used for producing a model. The time required for printing as well as the preparation and completion of the valve prototype was about four hours.

The printing process involved laying down successive layers of liquid photopolymer and curing the material with UV light emitted by lamps located on both sides of the printing head. During the printing process, two materials are sprayed onto the build tray: the model material and the support material. The thickness of a single photopolymer layer, along the vertical axis (z), depends on the operation mode, and it is 16 and 32 μm for the High Quality and the High Speed modes, respectively.

Table 1 shows the key properties of the 3D printing material-FullCure720 [27].

Although the rapid prototyping method used in this study is a high-precision method, the prototype was subjected to additional finishing operations, including calibration of the sleeve where the spool moves, and tapping to obtain threads in the valve body to mount pneumatic fittings and control elements. After all the elements were assembled and the valve body and the subbase were sealed with silicone paste, the prototype of the pneumatic valve was ready for testing.

## 6. Experimental Testing

The aim of the experimental work described here was to determine the flow-rate characteristics and flow parameters, C and b, of directional control valves with main elements produced by additive layer manufacturing.

Even though the relevant standard came into effect in 2013, the flow-rate characteristics presented in this article were determined with a test stand based on the previous version of the standard (ISO 6358-1989 Pneumatic fluid power. Components using compressible fluids. Determination of flow-rate characteristics).

**Figure 11:**
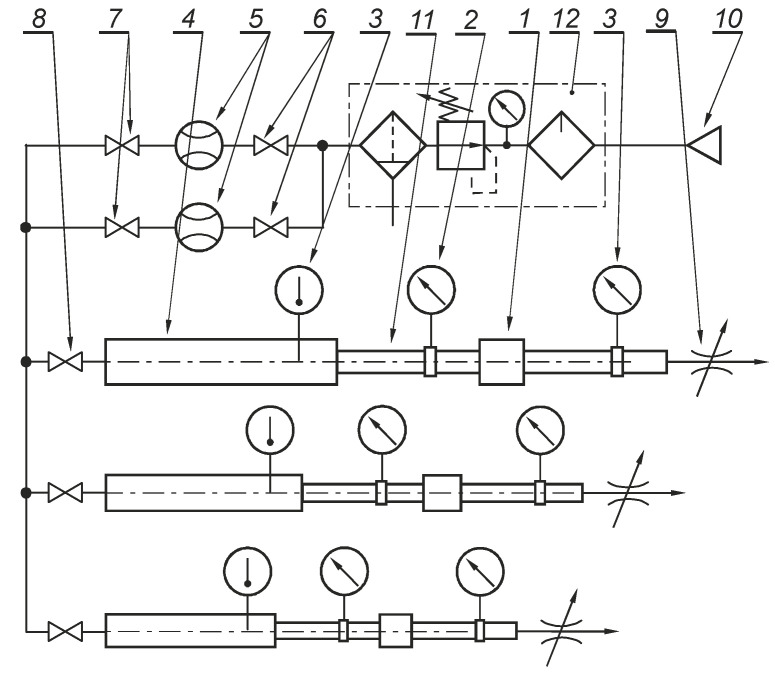
Diagram of the test stand for determining flow-rate characteristics.

The flow-rate characteristics of the pneumatic directional control valve were measured using a special test stand shown in Figure 11.

The test stand consists of: a valve tested (1), an upstream pressure gauge or transducer (2), a temperature-measuring instrument (3), a temperature-measuring tube (4), a flow-rate measuring device (5), shut-off valves (6, 7 and 8), a flow-control valve (9), a compressed gas source (10), a pressure-measuring tube (11), and a pneumatic filter-regulator-lubricator (FRL) unit (12).

**Figure 12:**
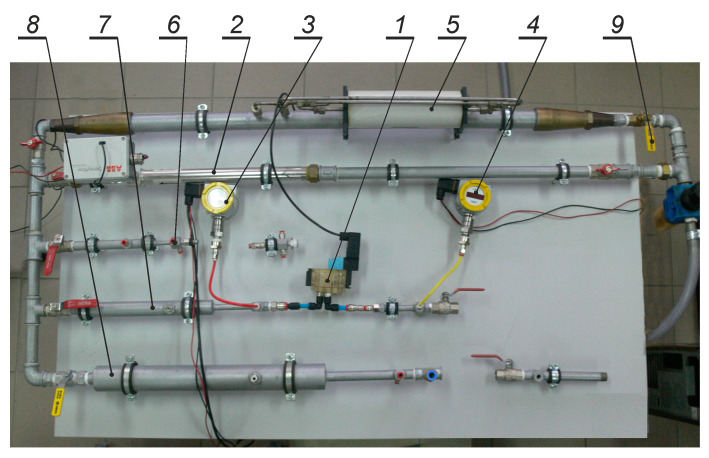
General view of the test stand: 1–valve tested, 2–Sensyflow D flow-rate measuring device, 3, 4–manometer, 5–U70 flow-rate measuring device, 6–measurement tube G1/8”, 7–measurement tube G¼”, 8–measurement tube G½”, 9–shut-off valve.

A general view of the test stand is given in Figure 12.

The ISO 6358-1989 standard defines the testing of pneumatic drive elements through which compressible fluids (gases) flow. The standard describes two testing methods, according to the type of element tested. One is used for flow-through type elements with the inlet and outlet ports. The other method is employed for flow-out type elements, i.e., those where air flows out into the atmosphere.

Two groups of flow parameters were distinguished: one comprising the sonic conductance, *C*, and the critical pressure ratio, *b*, and the other comprising the effective area (cross-sectional area of the inner diameter of the pressure-measuring tube), *A*, and the compressibility factor, *s*.

Group one parameters *C* and *b* are used to compare similar elements or calculate the pressure loss at an element and the flow through this element.

The critical pressure ratio *b* is the highest value of the pressure ratio *p*_1_/*p*_2_ at which the critical flow takes place in the element tested.

Parameter *C*, the sonic conductance, is the ratio of the mass gas flow q_m_* to the product of the inlet pressure *p*_1_* and the gas density *ρ*_0_ under normalized reference atmosphere conditions. The parameter is calculated for the critical flow rate at which the temperature of the gas at the inlet *T*_1_* is equal to the ambient temperature *T*_0_.

(7) $$C=\frac{q_{m}^{*}}{p_{1}^{*}\rho_{0}}\;\mathrm{for}\;T_{1}=T_{1}^{*}=T_{0}$$

where:

*C*–sonic conductance $\left[\frac{s\;m^{4}}{kg}\right]$,

$q_{m}^{*}$–mass flow rate $\left[\frac{kg}{s}\right]$,

$p_{1}^{*}$–inlet pressure [Pa],

$\rho_{0}$–gas density $\left[\frac{kg}{m^{3}}\right]$,

$T_{1}^{*}$–inlet temperature [K],

$T_{0}$–ambient temperature [K].

**Figure 13:**
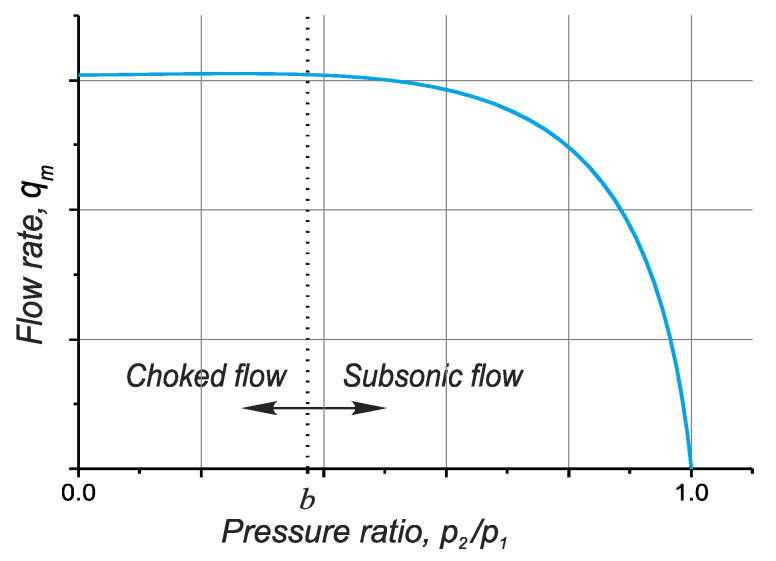
Theoretical flow-rate characteristic according to ISO 6358 and JIS B 8390.

From the experimental data it is evident that, for most elements used in pneumatic drives, the above model of phenomena describing the relationship between the mass flow rate and the pressure ratio can be illustrated as a quarter of an ellipsis and a horizontal line tangential to it, as shown in Figure 13.

When the gas flow rate reaches a local speed of sound in a certain cross-section of the pneumatic element, the mass flow rate at the inlet remains almost stable if the parameters are constant,. However, when the flow rate is smaller, the curve showing the mass flow rate *q_m_* as a function of *p*_2_/*p*_1_ is nearly elliptic (Figure 13).

## 7. Discussion and Comparison of Results

**Figure 14:**
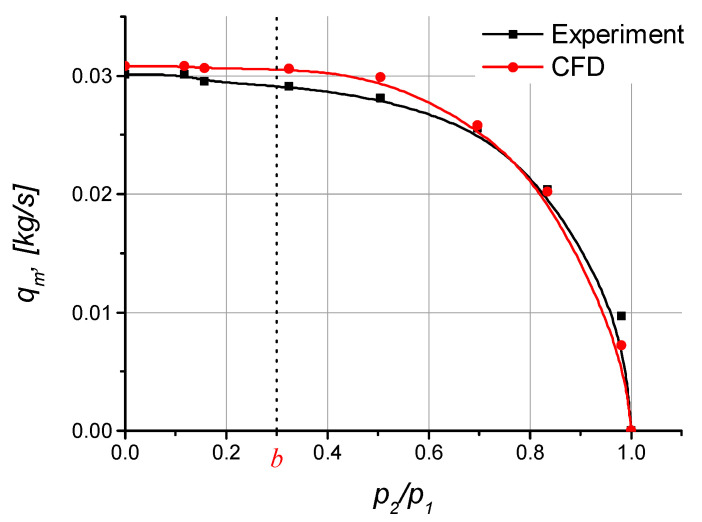
Mass flow rate vs. the pressure ratio.

To verify and validate the simulation and experimental results concerning the 3/2 pneumatic directional control valve, it was necessary to determine the relationships between the mass flow rate *q_m_* and the pressure ratio *p*_2_/*p*_1_. The results are shown in Figure 14.

The results obtained by mathematical modelling shown in the form of curves demonstrate good agreement with the experimental data. The small differences may result from the fact that the CFD modelling did not take into account either the surface roughness in the valve ports or the inner shape of the quick-connect pneumatic fittings.

The experimental and simulation results were used to determine the flow parameters, i.e., the critical pressure ratio b and the sonic conductance *C*.

According to the diagram in Figure 14, the critical pressure ratio *b* is 0.3. The sonic conductance *C* was calculated using relationship (7).

(8) $$C=\frac{q_{m}^{*}}{p_{1}^{*}\rho_{0}}=4.01849\cdot 10^{-8}\left[\frac{s\cdot m^{4}}{kg}\right]$$

The two parameters are characteristic of this type of valve. Further research is essential, however, because the values obtained in the study may be slightly different from those determined for a valve produced in a conventional way.

The method of development of pneumatic directional control valves proposed in this article has both advantages and disadvantages. The drawbacks include the printing technique, which may result in certain shape errors. Another shortcoming is surface roughness in areas where the support has been removed, because it affects the fluid flow through the valve ports. Obviously, there are many other challenges to overcome. The key benefit of this approach, however, is much shorter development times. The process from an idea to a finished product is now much faster. It is also possible to eliminate errors during the early stages of design. The combined use of CFD computer simulations and additive manufacturing enhances the design and analysis opportunities in the area of pneumatic directional control valves.

## 8. Conclusions

Currently, engineers are looking for methods that enable quick development of new innovative designs for immediate fabrication or optimization of the existing solutions to achieve the highest possible performance and energy efficiency. In pneumatic systems, energy efficiency can be improved mainly by reducing pressure loss and preventing leakage. As these factors are responsible for high operation and maintenance costs, changes in the design and production of pneumatic elements are necessary. The rapid prototyping technique used in this study has proved suitable to create 3D models and construct fully functional prototypes of pneumatic directional control valves in a short time and at a low cost. This technology can be used to produce customized pneumatic directional control valves. The approach presented in this paper enables us to obtain pneumatic valves with optimal design and performance, which can be produced in short series. The material that the pneumatic directional control valve was made of showed no defects after pressure and flow tests performed using a special test stand.

In this study, the 3D designed and printed valve had no seals. The subbase and the valve body were fastened with bolts. Silicone paste was also used to eliminate external leaks, i.e., leaks of compressed air into the atmosphere. The design of the spool enabled optimal fit between the spool and the subbase. This solution significantly reduced internal leaks, i.e., those between the ports, but it did not eliminate them completely. A better alternative would be to use seals on the spool. But this would require additional elements, i.e., seals, made in technologies other than 3D printing. The aim of this study was to use one process to construct a ready-to-use valve suitable for various industrial applications. In their future research, the authors will attempt to design and print a new type of valve with better flow parameters using a different technology, e.g., SLM.

## Figures and Tables

**Table 1:** Basic properties of FullCure720 (RGD 720).

	ASTM	UNITS	METRIC	UNITS	IMPERIAL
Tensile strength	D-638-03	MPa	50–65	psi	7250–9450
Elongation at break	D-638-05	%	15–25	%	15–25
Modulus of elasticity	D-638-04	MPa	2000–3000	psi	290,000–435,000
Flexural Strength	D-790-03	MPa	80–110	psi	12,000–16,000
Flexural Modulus	D-790-04	MPa	2700–3300	psi	390,000–480,000
HDT, °C @ 0.45 MPa	D-648-06	°C	45–50	°F	113–122
HDT, °C @ 1.82 MPa	D-648-07	°C	45–50	°F	113–122
Izod Notched Impact	D-256-06	J/m	20–30	ft Ib/inch	0.375–0.562
Water Absorption	D-570-98 24hr	%	1.5–2.2	%	1.5–2.2
Tg	DMA, E»	°C	48–50	°F	118–122
Shore Hardness (D)	Scale D	Scale D	83–86	Scale D	83–86
Rockwell Hardness	Scale M	Scale M	73–76	Scale M	73–76
Polymerized density	ASTM D792	g/cm^3^	1.18–1.19		
Ash content	USP281	%	0.01–0.02	%	0.01–0.02

## Data Availability

Not applicable.

## References

[1] Kheirollahi H., Abbaszadeh F. (2011). Application of rapid prototyping technology in dentistry. Int. J. Rapid Manuf..

[2] Soe S.P., Eyers D.R., Jones T., Nayling N. (2012). Additive manufacturing for archaeological reconstruction of a medieval ship. Rapid Prototyp. J..

[3] Lantada A.D., Morgado P.L. (2012). Rapid prototyping for biomedical engineering: Current capabilities and challenges. Annu. Rev. Biomed. Eng..

[4] Keating S.J., Gariboldi M.I., Patrick W.G., Sharma S., Kong D.S., Oxman N. (2016). 3D Printed Multimaterial Microfluidic Valve. PLoS ONE.

[5] Padash M., Enz C., Carrara S. (2020). Microfluidics by Additive Manufacturing for Wearable Biosensors: A Review. Sensors.

[6] Salmi M., Akmal J.S., Pei E., Wolff J., Jaribion A., Khajavi S.H. (2020). 3D Printing in COVID-19: Productivity Estimation of the Most Promising Open Source Solutions in Emergency Situations. Appl. Sci..

[7] Ruiz C., Kadimisetty K., Yin K., Mauk M.G., Zhao H., Liu C. (2020). Fabrication of Hard-Soft Microfluidic Devices Using Hybrid 3D Printing. Micromachines.

[8] Cavallo L., Marcianò A., Cicciù M., Oteri G. (2020). 3D Printing beyond Dentistry during COVID 19 Epidemic: A Technical Note for Producing Connectors to Breathing Devices. Prosthesis.

[9] Gardan N., Schneider A. (2015). Topological optimization of internal patterns and support in additive manufacturing. J. Manuf. Syst..

[10] Santana H.A., Amorim Júnior N.S., Ribeiro D.V., Cilla M.S., Dias C.M. (2021). 3D printed mesh reinforced geopolymer: Notched prism bending. Cem. Concr. Compos..

[11] Hopkinson N., Dickens P. (2001). Rapid prototyping for direct manufacture. Rapid Prototyp. J..

[12] Bochnia J., Blasiak S. (2019). Fractional relaxation model of materials obtained with selective laser sintering technology. Rapid Prototyp. J..

[13] Borić A., Kalendová A., Urbanek M., Pepelnjak T. (2019). Characterisation of Polyamide (PA)12 Nanocomposites with Montmorillonite (MMT) Filler Clay Used for the Incremental Forming of Sheets. Polymers.

[14] Schmid M., Amado A., Wegener K. (2014). Materials perspective of polymers for additive manufacturing with selective laser sintering. J. Mater. Res..

[15] Patalas-Maliszewska J., Topczak M., Kłos S. (2020). The Level of the Additive Manufacturing Technology Use in Polish Metal and Automotive Manufacturing Enterprises. Appl. Sci..

[16] Saini J.S., Dowling L., Kennedy J., Trimble D. (2020). Investigations of the mechanical properties on different print orientations in SLA 3D printed resin. Proc. Inst. Mech. Eng. Part C J. Mech. Eng. Sci..

[17] Gurr M., Mülhaupt R., Matyjaszewski K., Möller M. (2012). 8.04—Rapid Prototyping. Polymer Science: A Comprehensive Reference.

[18] Takagishi K., Umezu S. (2017). Development of the Improving Process for the 3D Printed Structure. Sci. Rep..

[19] Szczepański Ł., Pilarczyk W., Ambroziak A. (2019). Characteristics of Titanium Alloys used in the SLM Additive Technology. Biuletyn Instytutu Spawalnictwa w Gliwicach.

[20] Nazir A., Jeng J.-Y. (2020). A high-speed additive manufacturing approach for achieving high printing speed and accuracy. Proc. Inst. Mech. Eng. Part C J. Mech. Eng. Sci..

[21] O’ Connor H., Dickson A., Dowling D. (2018). Evaluation of the mechanical performance of polymer parts fabricated using a production scale multi jet fusion printing process. Addit. Manuf..

[22] Denoual M., Mognol P., Lepioufle B. (2005). Vacuum casting, a new answer for manufacturing biomicrosystems. Proc. Inst. Mech. Eng. Part B J. Eng. Manuf..

[23] Cazón A., Morer P., Matey L. (2014). PolyJet technology for product prototyping: Tensile strength and surface roughness properties. Proc. Inst. Mech. Eng. Part B J. Eng. Manuf..

[24] Takosoglu J.E., Laski P.A., Blasiak S. Innovative modular pneumatic valve terminal with self-diagnosis, control and network communications. Proceedings of the 20th International Conference on Engineering Mechanics (EM).

[25] Laski P.A. (2019). Fractional-order feedback control of a pneumatic servo-drive. Bull. Pol. Acad. Sci. Tech. Sci..

[26] Takosoglu J. (2020). Angular position control system of pneumatic artificial muscles. Open Eng..

[27] PolyJet Materials Data Sheet. https://3dprinting.co.uk/wp-content/uploads/2016/11/Polyjet-Materials-Data-Sheet.pdf.

